# Anti-malarial medicine quality field studies and surveys: a systematic review of screening technologies used and reporting of findings

**DOI:** 10.1186/s12936-017-1852-6

**Published:** 2017-05-15

**Authors:** Mirza Lalani, Freddy Eric Kitutu, Siân E. Clarke, Harparkash Kaur

**Affiliations:** 1 London School of Hygiene and Tropical Medicine, London, UK; 2 Makerere University, Kampala, Uganda; 3 Uppsala University, Uppsala, Sweden

**Keywords:** Antimalarial medicines, Medicine quality, Screening technologies, Medicine quality field surveys

## Abstract

**Background:**

Assessing the quality of medicines in low-middle income countries (LMICs) relies primarily on human inspection and screening technologies, where available. Field studies and surveys have frequently utilized screening tests to analyse medicines sampled at the point of care, such as health care facilities and medicine outlets, to provide a snap shot of medicine quality in a specific geographical area. This review presents an overview of the screening tests typically employed in surveys to assess anti-malarial medicine quality, summarizes the analytical methods used, how findings have been reported and proposes a reporting template for future studies.

**Methods:**

A systematic search of the peer-reviewed and grey literature available in the public domain (including national and multi-national medicine quality surveys) covering the period 1990–2016 was undertaken. Studies were included if they had used screening techniques to assess the quality of anti-malarial medicines. As no standardized set of guidelines for the methodology and reporting of medicine quality surveys exist, the included studies were assessed for their standard against a newly proposed list of criteria.

**Results:**

The titles and abstracts of 4621 records were screened and only 39 were found to meet the eligibility criteria. These 39 studies utilized visual inspection, disintegration, colorimetry and Thin Layer Chromatography (TLC) either as components of the Global Pharma Health Fund (GPHF) MiniLab^®^ or as individual tests. Overall, 30/39 studies reported employing confirmatory testing described in international pharmacopeia to verify the quality of anti-malarials post assessment by a screening test. The authors assigned scores for the 23 criteria for the standard of reporting of each study.

**Conclusions:**

There is considerable heterogeneity in study design and inconsistency in reporting of field surveys of medicine quality. A lack of standardization in the design and reporting of studies of medicine quality increases the risk of bias and error, impacting on the generalizability and reliability of study results. The criteria proposed for reporting on the standard of studies in this review can be used in conjunction with existing medicine quality survey guidelines as a checklist for designing and reporting findings of studies. The review protocol has been registered with PROSPERO (CRD42015026782).

**Electronic supplementary material:**

The online version of this article (doi:10.1186/s12936-017-1852-6) contains supplementary material, which is available to authorized users.

## Background

Malaria remains a major public health concern in low and middle income countries (LMICs), although, in recent years, there has been an overall decline in malaria incidence due to application of improved strategies for prevention, control and treatment [1]. The advent of artemisinin-based treatment has contributed to the reduction in disease transmission, with 79 out of 88 malaria-endemic countries having adopted artemisinin-based combination therapy (ACT) as first-line treatment for uncomplicated *Plasmodium falciparum* malaria by 2015 [2]. Assuring the quality of ACTs and other anti-malarials used to counter malaria is paramount in ensuring that the success of malaria prevention and control strategies is maintained. Yet, the reported finding that a third of anti-malarial medicines from malaria endemic countries failed chemical content analysis is a source of substantial concern, potentially threatening progress in control [3]. At the patient level, poor quality anti-malarials may result in treatment failure, leading to prolonged or severe illness and even death, as sub-therapeutic medicine concentrations increase the risk of recrudescence of malaria infection [4]. At the provider level this increases burden on already limited resources and undermines confidence in health providers [5]. From a public health perspective medicines with low stated active pharmaceutical ingredients (SAPI) or low bioavailability may select for drug resistant parasites [6]. An association between the quality of artemisinin-based medicines and drug resistance has been postulated but not as yet proven [7].

In-country, medicine quality can most readily be assessed at two stages in the supply chain; at point of entry and point of care. Firstly, anti-malarials permitted to enter the official supply chain in LMICs should, ideally be restricted to those produced by World Health Organization (WHO) prequalified manufacturers that have attained accreditation for good manufacturing practice (GMP) [8]. However, this is seldom the case, and often National Medicines Regulatory Authorities (NMRAs) will permit non-WHO prequalified medicines, assuming they have met GMP standards. Some countries may also have anti-malarials on the market that may not have been registered with the NMRA. Secondly, wholesalers have to obtain authorization from the NMRA before they can distribute medicines [9], and the products they import should satisfy the national regulatory requirements for obtaining pre-marketing authorization. Finally, subsequent medicine batches may undergo routine lot-quality sampling [10] and testing by a NMRA at the point of entry in some countries. However, the source of anti-malarials often varies, ranging from international wholesalers to direct donations from external organizations or medicine manufacturers [11]. Donated medicines can sometimes bypass these initial checks, making verification of their quality more challenging. Indeed, studies have shown that some donated medicines are more likely to be close to their manufacturer expiry date or have exceeded their shelf-life [12].

Point of entry sampling and analysis requires substantial initial and recurrent investment in resources, expertise and equipment, all of which are rarely affordable or available to LMICs on a routine basis. Thus, NMRAs most frequently rely on post marketing surveillance through periodic medicine quality sampling surveys at the point of care, using screening technologies [13]. Even so, the proportion of NMRAs in LMICs with regular access to screening technologies is not known. To this end, several new screening technologies have been developed in recent years. This review aims to present an overview of the screening tests used to assess medicine quality at point of care. It will also focus on the screening technologies and survey methods that have been used to assess medicine quality in field surveys, and the standard of reporting. A template for future reporting of studies is also proposed and is used to score the anti-malarial medicine quality studies included in this review.

### Principles of assessing medicine quality

**Fig. 1:**
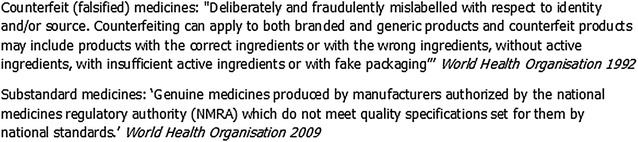
Definitions of poor quality medicines. *As of January 2017, the WHO member state mechanism has recommended that the use of “substandard/spurious/falsely-labelled/falsified/counterfeit medical products” should be replaced with “substandard and falsified medical products”

Tests for medicine quality are based on assessment of identity, chemical assay, disintegration and dissolution (bioavailability). These four core ‘principles’ provide the basis for medicine quality analytical technologies, which can also be further categorized as screening and confirmatory functions in a medicine quality surveillance system (MQSS) [14]. Fundamentally, an ideal test should be capable of detecting both counterfeit (or falsified) and substandard medicines (see Fig. 1 for definitions) through verification of the chemical content in terms of the stated active pharmaceutical ingredients (SAPIs) [15–17].

Surveillance systems for medicine quality in LMICs employ screening techniques and devices as the first stage for medicine quality analysis. Some of these technologies (listed in Table 1 together with their description, cost (where available) and role in the MQSS) are portable, simple to use and relatively inexpensive, making them suitable for screening large volumes of medicines. Nevertheless, they may only provide an indication of medicine quality, necessitating subsequent confirmation and quantification of the SAPI with more specific quantitative techniques found in medicine quality control laboratories, following methods described in international pharmacopeia.

**Table 1:** Tests for assessing the quality of a medicine

Test dimensions	Description	Medicine analytical technology	Role in a MQSS	Cost^a^
Identity	Verifies identity of SAPI	Visual examination of packaging Counterfeit detection device (CD3) RAMAN spectroscopy (hand-held device) Specialized mass spectrometry (MS) techniques Direct analysis in real time (DART) Desorption electrospray ionization—(DESI)	Screening Screening Screening Confirmatory	n/a * ** ***
Assay	Detection and quantitation of SAPI	Semi-quantitative thin layer chromatography (TLC) (GPHF MiniLab^®^) High performance liquid chromatography (HPLC) and coupled to mass spectrometer (LC–MS) Mass spectroscopy (MS)	Screening Confirmatory Confirmatory	* ** ***
Disintegration	Determines that a tablet or capsule will disintegrate	GPHF MiniLab^®^	Screening	*
Dissolution	Proxy measure of the bioavailability of a medicine (extent to which medicine will dissolve in the body)	Dissolution apparatus	Confirmatory	**

^a^Denotes relative cost of the technology; inexpensive—less than $10,000 (*), moderate—$10,000–$100,000 (**), very expensive—greater than $100,000 (***) [24]

Laboratory methods such as high performance liquid chromatography (HPLC) and mass spectrometry (MS) are needed to confirm the chemical content of a medicine in terms of its SAPI and are regarded as the ‘gold standard’ technique for medicine quality analysis. HPLC detects the SAPI and its amount is determined from the calibration curve measuring the peaks achieved using increasing known amounts of the reference standard. Thereafter, an analyst will assess if a medicine falls within tolerance limits for content in accordance with international pharmacopeia. Dissolution tests to determine the bioavailability of the medicine require specialist apparatus and can only be carried out when authorized pharmacopeia monographs exist. These techniques incur a high capital and maintenance cost and require specific laboratory infrastructure including highly skilled individuals for effective operation. Not all LMICs have the human and capital resources to thus maintain a fully functional MQSS. To enable medicines quality control laboratories to conform to international standards, the WHO operates a prequalification programme providing accreditation through ISO (ISO/IEC17025) certification. To achieve accreditation a medicines quality control laboratory must satisfy requirements for ‘Good practices for pharmaceutical quality control laboratories’, which will provide confidence in the services they provide [18].

In LMICs medicine quality screening technologies (Table 2) thus play a pivotal role in the surveillance of the quality of anti-malarials due to limited technical capacity. Analytical equipment such as HPLC and dissolution testing apparatus may be absent or only available at the national reference laboratory and even then, all pharmacopeia methods may not be possible to set up due to interrupted power supply, prohibitive costs involved (HPLC columns, reference standards etc.) and the requirement for highly skilled chemists to carry out the analysis. In contrast, portable screening techniques, require no electricity or advanced expertise to perform, can be used for testing large numbers of medicines in field surveys, and identifying suspect samples which are then subjected to comprehensive content analysis using confirmatory tests. Screening tests can thus be used as part of a national MQSS, most suitable for use in peripheral laboratories, border posts and other points of entry into a country.

**Table 2:** Descriptions for screening and confirmatory tests

Type of test	Description	Examples of tests
Screening	Basic tests for quality based on, but not restricted to, chromatographic and spectrometric techniques with the addition of visual inspection	GPHF MiniLab^®^; Raman handheld spectroscopy; counterfeit detection device (CD3)
Confirmatory	Methods listed in pharmacopeia, that can only be conducted in a laboratory by trained personnel and the results from which are quantifiable	HPLC; Dissolution; MS; LC–MS

### Guidelines for conducting medicine quality studies and surveys

Currently, there are no universally agreed guidelines on the study design and reporting of medicine quality studies or surveys, although there are at least three primary sources of information and guidance available. The United States Pharmacopeia (USP) operates a Promoting the Quality of Medicines Program providing technical and logistical support to LMICs. In 2006, USP published guidelines for sampling and analysing medicines for their quality [19]. In the same year Global Health Pharma Fund (GPHF) also produced a manual of testing procedures for the quality of medicines, to accompany the MiniLab^®^. The manual is updated on an annual basis to include testing guidelines for new medicines and modified procedures for existing medicines [20]. Lastly, in 2009, a proposal for a checklist for the sampling of medicines for medicine quality studies and surveys called MEDQUARG, was published [21].

## Methods

A systematic review of the published literature between 1990 and 2016 was undertaken in January 2016 and updated in October 2016. PubMed, Web of Science and Google Scholar were searched using predefined search terms as described in the protocol in Table 3. This review was carried out in accordance with PRISMA guidelines [22] and the protocol has been registered with PROSPERO, (ref: CRD42015026782). Articles were imported into Endnote and duplicates removed. Further searches were conducted using ancestral and forward citation of two prominent anti-malarial medicine quality reviews as well as searches of the grey literature using the Worldwide Antimalarial Resistance Network (WWARN) medicine quality surveyor [3, 23]. Where accessible, published national medicine quality surveys and reports were also obtained.

**Table 3:** Summary of review search criteria

Databases	1. PubMed 2. Web of Science 3. Google Scholar
Other sources	1. Nayyar et al. [23] 2. Tabanero et al. [3] 3. USP DQI country reports [85] 4. WWARN anti-malarial medicine quality surveyor [86]
Key search terms	Medicine OR drug quality AND survey OR screening Screening: AND poor quality OR counterfeit OR substandard (medicine OR drug) Detection: AND poor quality OR counterfeit OR substandard (medicine OR drug)
Eligibility criteria	
Dates	1990–2016
Language	English, French
Location	Central, South and South East Asia, Sub-Saharan Africa, Central and South America, Pacific Islands
Article type	Scientific publications in international peer-reviewed journals and grey literature (reports and surveys)
Types of studies	Field surveys in which anti-malarial medicines were assessed for quality, using a screening technique
Screening technique and outcome	1. Screening techniques: tests based on but not restricted to chromatographic and spectrometric techniques with the addition of visual inspection or visual inspection alone 2. Outcome measure (medicine quality)
Exclusion criteria	1. Reviews/Commentaries/Conference Papers/Letters 2. High specification, non-portable technologies (as defined by Kovacs et al. [24] with LMIC score <4^b^) 3. Feasibility studies 4. Non-anti-malarial medicine assessed for quality alone 5. Only results of either screening or confirmatory tests presented when both undertaken
Search dates	January 2016, updated October 2016

^b^Kovacs et al. [24] have reviewed all medicine quality screening technologies in use and categorized them by cost and portability. Their scoring matrix has been used as an exclusion criterion with an LMIC score of less than 4 representing those technologies that are less feasible for use in LMICs due to their lack of portability and high cost

Titles, abstracts and executive summaries were assessed by the first author (ML) of this manuscript for their relevance (summarized in Table 3). Studies that reported utilizing screening tests for anti-malarials were included for full text review. Studies and reports published in English and French were included.

**Fig. 2:**
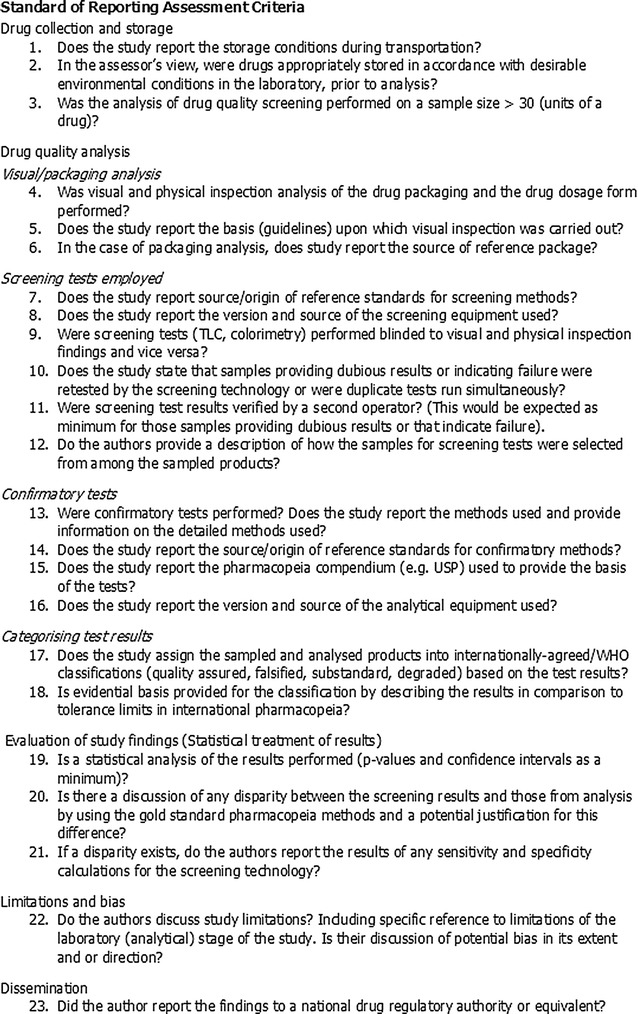
Criteria for assessing the standard of reporting of medicine quality surveys. Adapted from a previously published review, published procedures of individual tests, MEDQUARG guidelines, USP Medicine Quality and Information Program Guidelines and consideration of the GRADE guidelines (quality of evidence and strength of recommendations for diagnostic tests or strategies)

An assessment of the quality of reporting of the eligible reports and studies was undertaken to examine the rigour of study design and sources of potential bias in findings. The authors applied criteria adapted from a previously published review [25], published procedures of individual tests, USP Medicine Quality and Information Program Guidelines [19], MEDQUARG guidelines [21], and the GRADE guidelines (quality of evidence and strength of recommendations for diagnostic tests or strategies) [26, 27]. The process gave rise to a final list of 23 criteria of reporting quality (listed in Fig. 2); these items assessed how studies report aspects of field collection of medicine samples, storage, method of medicine quality assessment categorized as level I (visual inspection), level II (screening tests) and level III (confirmatory tests), medicine quality analysis and interpretation, dissemination, study limitations and bias. Data extracted from eligible studies were organized under the following headings; study details (year of collection and location), medicine sampling and storage, screening tests, confirmatory tests, classification of quality, statistical tests, limitations, bias and dissemination. Studies included at this stage were full text versions and were independently assessed by two of the authors (ML and FEK). Discrepancies were clarified and a final list of reconciled studies was produced for inclusion. Two separate tables were compiled, one listing studies that used a screening test only and studies using both screening plus confirmatory tests (see Additional files 1, 2). The scoring criteria was applied to both sets of studies (screening alone and screening plus confirmatory).

## Results

**Fig. 3:**
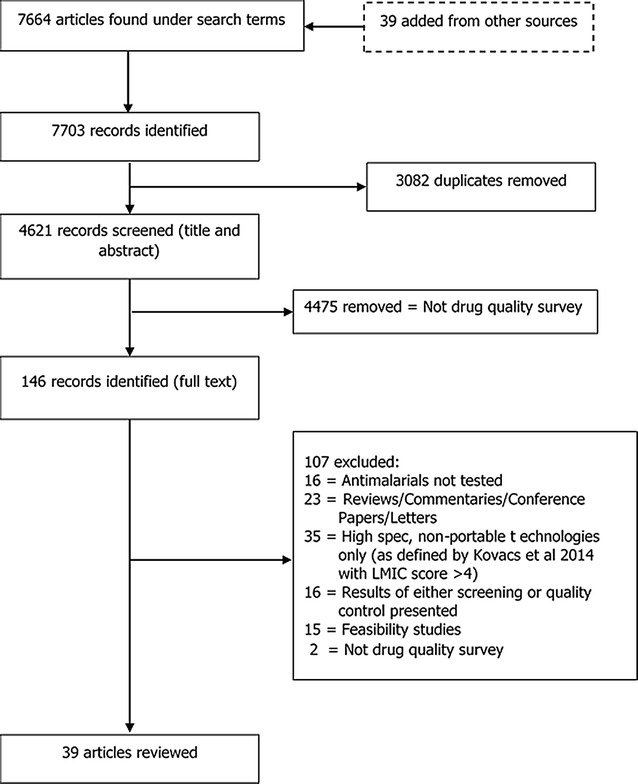
Literature review search strategy

The titles and abstracts of 4621 records were screened (after duplicates were removed) and 146 were identified based on the eligibility criteria (Fig. 3). Studies excluded at this stage were predominantly not related to medicine quality and included pharmacovigilance (drug safety) studies, diagnostic testing for illicit or banned substances and several clinical trials of new drug targets. The remaining 146 records were subjected to full text review against the exclusion criteria. This resulted in 39 articles that assessed the quality of anti-malarial medicines using a screening test which were subsequently reviewed and summarized.

### Types of studies

Of the 39 included studies and surveys in this review, the vast majority (36/39) were peer-reviewed articles published in international journals and three were non-peer-reviewed publications, which comprised two multi-country surveys (one conducted by the WHO [28] and the other by USP [29]) and a national medicine quality survey undertaken by the Malaria Control and Pharmacy and Poisons Board for Kenya [30]. The latter was the only national Ministry of Health agency survey report that met the eligibility criteria to be included in this review. Of the 39 included studies, 33 were conducted in South East Asia and Sub-Saharan Africa, with the remainder undertaken in Afghanistan [5], India [31, 32] Guyana and Suriname [33], the Amazon Basin [34] and Papua New Guinea [35]. Of the 25 studies from Africa, 15 were conducted in Nigeria and Ghana [28, 36–48]. The remainder comprized the aforementioned national medicine quality survey conducted by a Ministry of Health agency in Kenya [30] and two multi-country surveys, conducted by USP in Madagascar, Senegal and Uganda [29] and the WHO in Kenya, Nigeria, Ghana, Ethiopia, Tanzania and Cameroon [28]. Multi-country studies accounted for 12/39 (30.8%) [28, 29, 33, 34, 42–46, 49–51] that met the inclusion criteria.

A variety of outlets from all levels of the distribution chain were represented among the studies. Most of the studies sampled at point-of-care; public health facilities (government funded hospitals and clinics), private sector pharmacies and the informal sector (markets stalls, itinerant sellers and grocery shops). Three studies sampled from the highest level of the distribution chain such as wholesalers and central medical stores (in addition to hospitals, pharmacies etc.) [30, 52, 53]. In the included studies, a broad range of anti-malarial medicines were sampled and analysed for quality.

### Tests used for screening medicine quality

The WHO medicine testing guidelines recommends combining qualitative and quantitative approaches to analysis, to establish the identity, content and disintegration of a medicine [54]. Qualitative tests include visual inspection, colorimetric tests and tablet or capsule disintegration. Visual inspection involves assessment of the medicine packaging, patient information leaflet and the medicine itself. Misspellings, absence of an expiry date or batch number and obvious signs of deterioration of the product indicate a poor quality medicine [55]. However, for a full appraisal, prior knowledge of the authentic manufacturers packaging is required, which would not be routinely available to a patient or a medicine outlet proprietor. Colorimetric tests are identity tests that involve a simple colour reaction to verify presence of the SAPI. The disintegration test requires the tablet/capsule to disintegrate in water heated to 37 °C, within 30 min. If this does not occur, it could indicate a poor quality product. Thin layer chromatography (TLC) is an example of semi-quantitative testing [56]. The combination of the steps of visual inspection followed by disintegration testing give an assessment of deficiencies related to medicine solubility and availability. The third step of carrying out a colour reaction indicates if the SAPI is present before employing a TLC run for verification of whether the quantities of medicine claimed on the label are in the sample.

All of these tests are incorporated in the GPHF MiniLab^®^ which is capable of testing around 80 WHO essential medicines (including anti-malarials) and is reportedly available in over 90 countries worldwide, and often used in LMICs as an integral component of a MQSS [57]. TLC is an identity/content test that provides a semi-quantitative analysis in which a spot of the medicine under investigation is solubilized in an appropriate solvent and applied to a TLC plate and should migrate at the same rate as that for the similarly solubilized reference standard (equivalent to 80 and 100% of the SAPI). If the spot formed by the medicine is obviously different to the reference spots in colour and size this may indicate a poor quality sample. Indeed the sample spot must be at least similar to the lower working reference spot representing ‘80%’ to be considered as a ‘pass’ according to MiniLab^®^ guidelines [20]. Thus colorimetric tests and TLC constitute a subjective evaluation of medicine quality, dependent on the visual acuity of the technician conducting the test.

Other tests intended for use in field surveys for detecting poor quality medicines include paper test cards, the Raman handheld device and near-infrared spectrometers (NIR), all of which are in various stages of development [58, 59]. These tests employ spectroscopy or separation techniques and are based on the principle of identity, verifying the SAPI in a medicine sample. The Raman and NIR spectrometer devices scan medicine samples through the blister pack. They identify a unique spectral ‘fingerprint’ allowing comparison of a suspect sample with a genuine medicine which requires access to a library of spectra for each individual brand of medicine on the market. Thus far, only the TruScan^®^ handheld Raman device has demonstrated the ability to detected counterfeits the field [60]. In contrast to the Raman device, NIR can distinguish whether the excipients in a medicine sample are in the correct proportions, suggesting that the medicine is falsified, but it cannot detect substandard medicines [61]. Separation techniques employing paper-based chromatography allow testing of multiple SAPIs on a single piece of card (known as multiplexing) [62]. They are inexpensive and simple to use but have a low accuracy in terms of quantification of the SAPI. Field experience of the Raman, NIR and paper tests cards to date is restricted to a limited number of studies [46, 60, 63]. The cost of the Raman device remains a limiting factor to more widespread use. Nevertheless, a new, comparatively low cost and portable prototype version of NIR has recently demonstrated a promising capability in detecting falsified samples of ACTs and artemisinin monotherapies [64].

Amongst the 39 eligible field surveys of medicine quality published between 1990 and 2016, there was limited variation in the screening techniques that had been employed. All studies had utilized visual inspection, disintegration, colorimetry and TLC either as components of the GPHF MiniLab^®^ or as individual tests. Overall, 4/39 (10%) studies were limited to visual inspection alone [33, 36, 49, 65]. In 7/39 (18%) studies, more than one screening test was employed; of which, five compared the MiniLab^®^ with Raman Spectrometry and/or near infra-red (NIR) [43–46, 66]. The CD-3 device, which analyses medicine packaging was reported in just one study [67] and another study employed MVHimagePCv8.exe Color Software which measures colour intensity of samples subjected to the colorimetric Fast Red TR test, using digital imagery [47].

### Standard of reporting

**Fig. 4:**
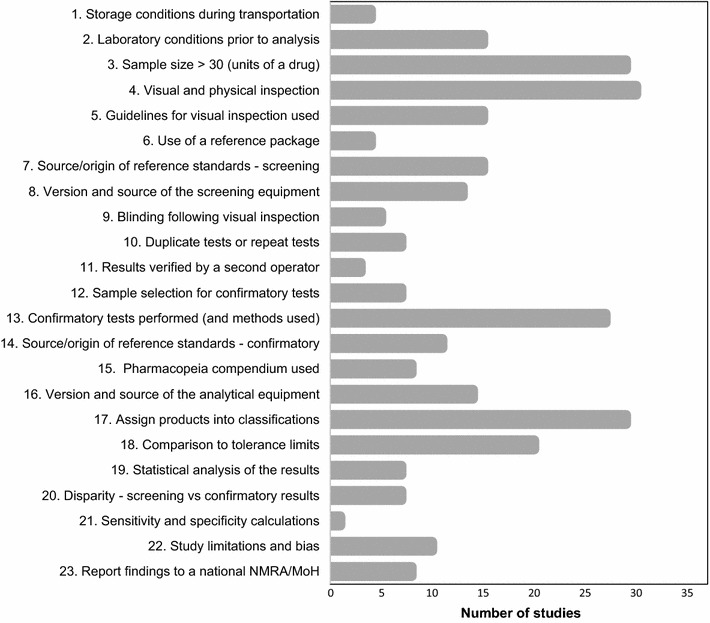
Number of medicine quality survey reports satisfying each reporting quality criterion

In addition to the variation between studies in the outlets from which medicines were collected for analysis and the screening techniques employed, there was considerable variation in how results were reported. None of the included studies met all 23 criteria of the standard of reporting (see Fig. 4). Scores ranged from 17/23 (74%) as the highest [67] and 2/23 (9%) [47] as the lowest. Studies published before 2006 [10/39, (26%)] scored an average of 6.9 compared to 8.5 for those published after 2006. This marginal improvement may have been in response to the publication of the MiniLab^®^ manual (instructions on testing procedures) and USP guidelines in 2006. The number of studies satisfying each of the quality of reporting assessment criteria are presented in Fig. 4.

### Medicine storage and collection

Improper storage conditions have been suggested as a possible risk factor for deteriorating medicine quality over time, which if drugs are not stored adequately prior to analysis, could cause the proportion of poor quality medicines to be overestimated (misclassification bias) [68]. Nevertheless, reporting of storage conditions after collection was generally poor; only 4/39 (10%) studies stated maintaining appropriate storage conditions during transit from the outlet to the laboratory and less than half (15/39 (39%)) reported storage conditions in the laboratory prior to analysis [30, 65, 67, 69]. The scale of the surveys and number of samples analysed varied substantially between studies. USP guidelines for medicine sampling state that a minimum of 30 dosage units per location be collected, sufficient to carry out testing for identity and content of SAPI and dissolution [19]. Yet overall, 10/39 (26%) studies based their findings on a sample size of less than 30 [32, 35, 37–41, 47, 49, 52].

### Visual inspection/packaging analysis

Visual assessment of medicine packaging and the tablet or capsule is an inexpensive approach but requires the original packaging from the manufacturer for accurate comparison. The GPHF MiniLab^®^ manual provides guidelines to standardize visual inspection. In this review 9/39 (23%) studies did not state undertaking a visual assessment [31, 32, 46, 47, 52, 70–72]. Of those studies that did undertake visual inspection, 12 mentioned referring to MiniLab^®^ guidelines [5, 28, 29, 34, 43, 45, 48, 66, 73–76]. Only 4/39 (10%) studies carried out packaging analysis (all from SE Asia) and reported comparison with a sample of the genuine packaging obtained from the medicine manufacturer [39, 65, 67, 77]. One study, explicitly stated contacting manufacturers for the original packaging without response [69].

### Description of laboratory procedures (screening tests)

References standards are pure chemical compounds obtained from chemical manufacturing companies and are used by pharmacopeia such as USP as a basis for their official monographs for analysts to adopt. This review found that 15/39 (39%) studies stated using reference standards in the analytical process and reported on the source of the standard [30, 35, 38–42, 45, 46, 49, 65, 67, 76, 78, 79]. Of the 35/39 (89.7%) studies (excluding those that conducted visual inspection alone), 10/35 (29%) did not mention the source of the screening tests (and reagents), but simply named the device they were utilizing; MiniLab^®^ or a Raman handheld device [28–32, 42, 45, 48, 51, 72].

Interpretation results from visual inspection, colorimetric and TLC tests are subjective and dependent on the visual acuity of the operator. A second operator to independently verify test results would enhance the validity of a study by minimizing the risk of operator error in results interpretation, particularly for the semi-quantitative TLC test in which there is a greater risk of misclassification bias. Overall, 31 studies used TLC, but only three reported using a second operator to verify results [38, 69, 75]. For 8/39 (21%) studies using visual inspection and/or colorimetry, a second operator would be unnecessary as results are more definitive in comparison to TLC.

USP guidelines for medicine quality testing using the MiniLab^®^, state that any samples of doubtful quality or ‘failed’ samples, as well as 5–10% of passed samples must be retested using disintegration and TLC techniques [19]. Devices such as the Raman handheld and CD3 are new additions to the market and at the time of the studies in which they undertaken, there was no mention of the need for test repetition. Of the studies that employed disintegration and TLC, just 7/30 (23%) reported repeat testing of samples [30, 43–45, 73, 75, 76]. Moreover, just 5/39 (13%) studies reported having blinded the operators to results from visual/packaging analysis or from screening tests, thus increasing the risk of performance (observer) bias [50, 51, 67, 70, 77]. In total, 7/39 (18%) studies discussed using a rudimentary strategy for selecting medicines from their initial sample to be analysed further by confirmatory tests (often to verify doubtful quality or failure of samples) [28–30, 67, 69, 74, 76]. The remaining studies either did not use confirmatory tests or the screening tests did not highlight any failing samples.

### Confirmatory testing using pharmacopeia methods

In total, 9/39 (23%) studies did not employ confirmatory tests, hence their results simply provide an indication of medicine quality [31, 43–48, 66, 78]. A fairly wide range of confirmatory tests were applied in the remaining 30/39 (77%) studies, although three studies did not specify the test that had been carried out [30, 34, 75]. Tests assessing both the physical and chemical properties of a medicine were undertaken. For the former, this included friability (the tendency for a tablet to break), uniformity of mass (weight variation amongst samples from the same batch) and hardness tests. Chemical tests included HPLC or Mass spectrometry (MS) (to assess content of the SAPI) or dissolution (medicine bioavailability). Of the 27 studies that reported the specific type of confirmatory tests they utilized, 12 conducted physical tests and also undertook chemical testing in parallel [32–35, 37, 38, 40, 41, 52, 72]. The remaining studies (15/27 (56%)) used chemical tests alone, either LC/MS or dissolution apparatus. Studies conducted in the last 5 years used confirmatory tests only, suggesting either that more institutions have now acquired HPLC and dissolution equipment or there is greater recognition of their importance as highly accurate techniques.

Only 11/30 (37%) studies employing confirmatory tests stated the source of the reference standards obtained [35, 38–42, 49, 51, 65, 76, 77] and just 14 stated the manufacturer of the analytical equipment [5, 32, 35–41, 49, 51, 52, 65, 77]. In total, 7/27 (26%) studies that stated a using a specific type of confirmatory testing did not report using an international pharmacopeia [30, 50–52, 69, 76, 77]. Reference to the use of a pharmacopeia reassures the reader that ratified methods for testing medicines were employed.

In this review, 7/39 (18%) studies did not categorize failing medicines, reporting them simply as having ‘failed the tests’ or ‘not compliant’ without providing further details of the criteria used [32, 34, 37, 43, 66, 73, 74]. The remaining studies classified failing samples as counterfeit, falsified, fake, substandard, degraded and poor quality either alone, or in combination. In the absence of guidelines, no criteria for determining degraded medicines were provided. There are currently no universally agreed definitions for poor quality medicines which may account for the wide array of terms used in the included studies [80]. Yet, standardization for definitions is important as it enables regulatory authorities to plan appropriate action to address the problem of each specific type of poor quality medicine.

Tolerance limits refer to the standards listed in medicine monographs in pharmacopeia for the SAPI of a medicine. Medicines failing to meet these standards either by having a sub-optimal amount of SAPI or too much, would be considered to be of poor quality. Of the 27 studies that stated the specific type of confirmatory test employed, nine did not state tolerance limits for the medicines and of these [29, 30, 33, 34, 50, 51, 69, 77] eight studies still categorized the failing samples as falsified, counterfeit, poor quality and/or substandard. The remaining study only stated that samples had ‘failed tests’ [34]. Without stating tolerance limits to compare the SAPI in the sample against pharmacopeia standards, these categorizations are unsubstantiated.

### Statistical treatment of results

A thorough analysis of obtained data should include a statistical analysis, including *p* values and/or confidence intervals which take account of the sampling variation in surveys, indicating the precision of any estimates obtained [81]. Of the included studies, only 7/39 (18%) [36, 50, 51, 65, 67, 69, 72] undertook a statistical analysis presenting their results with *p* values or confidence intervals.

Additionally, disparity between results from screening and confirmatory tests in terms of sensitivity and specificity of the screening test to classify medicines as poor quality, should be reported. For this study, the terms sensitivity and specificity are used in their statistical sense, as of a measure of the performance of binary classification tests differentiating between those medicines that are poor quality and good quality and how accurate these results are when compared to the ‘gold standard’ tests (HPLC and dissolution). Overall, 14/30 (47%) studies that stated using confirmatory tests, recorded a disparity with screening tests which had either overestimated or underestimated the quality of the samples [5, 28, 29, 32–34, 37–42, 49, 52, 67, 70, 71, 74, 76]. However, only 7/30 (23%) of these studies highlighted this disparity [5, 28, 34, 42, 67, 71, 76], and a sensitivity and specificity calculation was presented in just one study [28]. Thus, although authors have reported findings that demonstrate a lack of accuracy of the screening test, they have rarely described the reasons for inconsistency or as a minimum, drawn attention to the discrepancy.

### Limitations and bias

Of the included studies, 15/39 (39%) did not discuss any potential limitations of the study design, sampling strategy or laboratory methods used [30, 32, 33, 35, 37–41, 46–48, 50, 52, 78]. Of the remaining 24 studies, 10 provided an account of limitations specifically relevant to the type of screening test used, of which, five studies included a discussion of the disparity between results from screening and confirmatory tests [5, 28, 29, 34, 65, 67, 69, 71, 74, 75].

Overall 16/39 (41%) studies discussed a potential risk of bias in their studies. Of these, 15 studies mentioned the risk of selection bias related to either the sampling strategy, a small sample size or in the sample selection for analysis by confirmatory tests [5, 28, 29, 31, 34, 36, 65–67, 69, 71, 72, 74, 75, 77]. The risk of operator error in performing a test incorrectly or misinterpreting results (misclassification bias) was cited by 3/16 (19%) studies [65, 66, 74]. In addition, 2/16 (13%) studies stated a concern of performance (observer) bias and the need to blind any additional operators involved in testing (or re-testing) medicine samples [51, 67].

### Dissemination of findings

Overall, 9/39 (23%) studies stated that they had shared their findings (or intended to do so) with either the Ministry of Health or the relevant NMRA [28–30, 33, 36, 67, 69, 71, 73].

## Discussion

In contrast to the extensive discourse in recent years on how medicine quality should be defined, much less attention has been paid to reporting of both the technologies used to assess medicine quality and the results obtained [80]. This review summarizes the current evidence on the reporting of findings from anti-malarial medicine quality surveys in LMICs that have employed screening technologies and provides guidance on how reporting could be improved for future studies. Our results highlight the great variation in study design, survey methods and laboratory procedures used. The lack of standardization hinders comparison between studies, and is a potential source of error and bias. The review reveals the need for procedures to be more comprehensively reported in a standardized manner, to more readily evaluate the accuracy of estimates of medicine quality obtained in surveys, and to compare results across studies. Medicine quality surveyors and researchers ought to state in detail, the analytical methods which they have used and provide an indication of the reliability of the results obtained [82]. Clear and thorough reporting on analytical methods and study findings should include the following aspects as a minimum; sampling strategies, specific details of screening and confirmatory test procedures, test repetition, blinding of operators, use of reference standards, and reporting on risk of bias to enable results to be interpreted with greater confidence.

Establishing conclusive evidence for the accuracy of commonly used screening tests is challenging as there is little information available on the precision of methods for detecting poor quality anti-malarials. The accuracy of the most frequently employed screening technique, the MiniLab^®^ (TLC test), has been questioned as it has previously been shown to overestimate medicine quality for anti-malarials, producing false positive results when samples are compared to analysis by HPLC [5, 28, 83]. Indeed, the MiniLab^®^ has been described as “only being able to detect grossly substandard or counterfeit medicines” [71]. Nevertheless, cost effective screening technologies have a key role in providing an indication of medicine quality and are especially useful in settings where confirmatory tests are not readily available. Yet, medicine quality surveys that elect to solely utilize screening technologies should be scrutinized thoroughly and regulatory decisions actioned after completing verification of suspect samples by confirmatory tests [84].

This review, has found that just under half (14/30 (47%)) of the studies reported a discrepancy in the results once confirmatory testing had been carried out subsequent to using screening tests. However, only the WHO multi-country study carried out a specificity and sensitivity calculation to explore the extent of the discrepancy [28]. There is a need to establish the precise accuracy of each of the screening techniques available for all medicines they test, and it should be mandatory for manufacturers of new technologies to report the sensitivity and specificity of the test, determined through both feasibility testing in a laboratory setting and piloting in the field.

### Strengths and limitations of the review

The quality assessment score used in this review has suggested that reporting in anti-malarial medicine quality studies is not satisfactory; potentially limiting the ability of an NMRA to take action in the case of finding a poor quality medicine. However, there are two caveats to the criteria used. Firstly, if a study has not employed confirmatory tests it will inevitably be assigned a lower score as the study cannot fulfil 8 of the 23 criteria stipulated. Secondly, studies failing to provide pertinent information on the methods used, as well as those with a limited study design, would both obtain a low score. Nonetheless, this information is essential to have confidence in the accuracy of the results reported. A strength of the scoring criteria is that they do provide a broad indication of the rigour of the research design and the reliability of the results. The wide range in scores (2–17) in this review indicate that there is still considerable room for improvement in the reporting of medicine quality studies.

A review of the grey literature using keyword search terms in generic web search engines was not conducted. The majority of findings [36/39 studies (92%)] presented in this review are from academic peer-reviewed papers published in international journals and not from reports produced by NMRAs which are often the key organizations at country level conducting anti-malarial medicine quality surveys. Country specific medicine quality reports from national surveys conducted by NMRAs or similar agencies may exist but are less accessible, as they are either published internally or for dissemination to funders and operational partners and may only be published on an *ad hoc* basis. The limited number of these types of report found in the process of the literature search did not meet the inclusion criteria.

## Conclusions

The frequency with which medicine quality studies and surveys are being conducted by a diverse profile of organizations and academic institutions from LMICs, North America and parts of Europe has increased appreciably. Whilst a multidisciplinary approach to the field of medicine quality is both required and encouraged, researchers involved in these studies can differ in their disciplinary background (pharmacy, chemistry, medicine, epidemiology etc.), knowledge, and experience, placing an increased and urgent need for convergence toward an agreed approach to studies and surveys [84].

This review has found much heterogeneity across the included studies in terms of study design and consistency in reporting, which impacts on the generalizability of survey results and further perpetuates the lack of information on the accuracy of the most popular screening technologies. The introduction of reporting guidelines, such as the CONSORT and STROBE guidelines, have helped to standardize the reporting of clinical trials and epidemiological studies, and increased the clarity and scientific rigour of study design, facilitating the interpretation of results and comparison between studies. In contrast, there is little guidance on the reporting of findings from medicine quality surveys with the sole exception of the MEDQUARG checklist, applied in only one of the studies included in this review [69]. The MEDQUARG checklist provides guidance on sampling in medicine quality surveys. At country level, USP have provided guidance for conducting medicine quality surveys. Despite the availability of these two sets of guidelines the divergence in study designs limits the interpretation of findings and the comparison between studies.

It has also been highlighted here that the standard of reports is limited by a number of common weaknesses across studies. This includes small sample sizes (especially at the level of the confirmatory test), a lack of blinding of operators, limited results verification, and ambiguity in sample selection for confirmatory tests, all of which may bias results. A set of standardized guidelines would help to reduce variation and decrease the risk of bias in medicine quality studies. The authors propose that the measures for reporting the quality of a medicine quality survey in this review, should be used in conjunction with the MEDQUARG and USP guidelines, as a checklist for academics and programme managers in designing surveys and reporting results. This would facilitate the assessment of the reliability and accuracy of findings by national and international authorities (NMRAs, WHO, USP etc.), journal editors and peer reviewers.

## Additional files

**Additional file 1.** Screening tests only. Included studies; screening tests only. Table representing studies that employed screening tests only that were included in the review and assessed for their standard using the criteria in the proposed template.

**Additional file 2.** Screening plus confirmatory. Included studies; screening tests plus confirmatory analysis. Table representing studies that employed screening tests plus confirmatory analysis that were included in the review and assessed for their standard using the criteria in the proposed template.

## Abbreviations

ACT: artemisinin-based combination therapy
CONSORT: Consolidated Standards for Reporting Trials
DQSS: drug quality surveillance system
NMRA: National Medicine Regulatory Authority
GMP: good manufacturing practice
GPHF: Global Health Pharma Fund
HPLC: high performance liquid chromatography
LMICs: low-middle income countries
NIR: near infrared
SAPI: stated active pharmaceutical ingredient
STROBE: the strengthening the reporting of observational studies in epidemiology
TLC: thin layer chromatography
WHO: World Health Organization
